# Characterization of self-anticipated pain score prior to elective surgery - a prospective observational study

**DOI:** 10.1186/s12871-021-01303-y

**Published:** 2021-03-19

**Authors:** Wei-Shu Chang, Yi-Ting Hsieh, Moa-Chu Chen, Shu-Ching Chang, Tzu-Shan Chen, Yun-Chi Chang, Yu-Chuan Tsai, Chen-Fuh Lam

**Affiliations:** 1 Department of Anesthesiology, E-Da Hospital and E-Da Cancer Hospital, Kaohsiung, Taiwan; 2 Department of Medical Research, E-Da Hospital and E-Da Cancer Hospital, Kaohsiung, Taiwan; 3 Department of Medical Imaging and Radiological Sciences, College of Medicine, I-Shou University, Kaohsiung, Taiwan; 4 School of Medicine, I-Shou University College of Medicine, Kaohsiung, Taiwan

**Keywords:** Numeric rating scale, Pain expectation, Pre-anesthesia assessment, post-anesthesia care unit, Surgery-related pain

## Abstract

**Background:**

Current principles of postoperative pain management are primarily based on the types and extent of surgical intervention. This clinical study measured patient’s self-anticipated pain score before surgery, and compared the anticipated scores with the actual pain levels and analgesic requirements after surgery.

**Methods:**

This prospective observational study recruited consecutive patients who received elective surgery in the E-Da Hospital, Taiwan from June to August 2018. Patients were asked to subjectively rate their highest anticipated pain level (numeric rating scale, NRS 0–10) for the scheduled surgical interventions during their preoperative anesthesia assessment. After the operation, the actual pain intensity (NRS 0–10) experienced by the patient in the post-anesthesia care unit and the total dose of opioids administered during the perioperative period were recorded. Pain scores ≥4 on NRS were regarded as being unacceptable levels for anticipated or postoperative pain that required more aggressive intervention.

**Results:**

A total of 996 patients were included in the study. Most of the patients (86%) received general anesthesia and 73.9% of them had a history of previous operation. Female anticipated significantly higher overall pain intensities than the male patients (adjusted odd ratio 1.523, 95% confidence interval 1.126–2.061; *P* = 0.006). Patients who took regular benzodiazepine at bedtime (*P* = 0.037) and those scheduled to receive more invasive surgical procedures were most likely to anticipate for higher pain intensity at the preoperative period (*P* < 0.05). Higher anticipated pain scores (preoperative NRS ≥ 4) were associated with higher actual postoperative pain levels (*P* = 0.007) in the PACU and higher total equivalent opioid use (*P* < 0.001) for acute pain management during the perioperative period.

**Conclusion:**

This observational study found that patients who are female, use regular benzodiazepines at bedtime and scheduled for more invasive surgeries anticipate significantly higher surgery-related pain. Therefore, appropriate preoperative counseling for analgesic control and the management of exaggerated pain expectation in these patients is necessary to improve the quality of anesthesia delivered and patient’s satisfaction.

**Supplementary Information:**

The online version contains supplementary material available at 10.1186/s12871-021-01303-y.

## Background

Inadequate postoperative pain management can lead to physical and psychological distress in patients as well as impact surgical wound healing [1–5] and increase the risk of developing postoperative delirium [6] and cardiopulmonary and thromboembolic events [7]. Although numerous clinical pathways and strategies have been implemented to improve postoperative pain management, such as the introduction of the enhanced recovery after surgery (ERAS) program and multimodal analgesia (MMA), rates of inadequate postoperative pain management remain as high as 40–56.4% in the general surgical population [8–11].

**Fig. 1:**
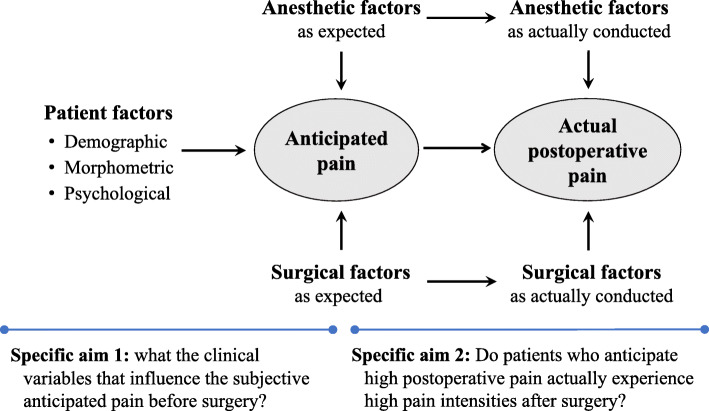
Specific aims of the study. The clinical variables that might be associated with increased anticipated pain were defined as patient’s characteristics, anesthesia-related and surgical related factors

Several perioperative factors such as age, catastrophic pain scores, gender, psychological distress, and operation type have been suggested to be closely associated with the postoperative pain intensity and analgesic usage [12–14]. It has been found that for breast cancer patients undergoing mastectomies or conserving surgeries, higher postoperative pain expectations and high preoperative distress can predict more intense postoperative pain [15, 16]. A prospective observational study conducted in females undertaking hysterectomies also showed that pre-surgical fears of the immediate consequences of surgery was associated with increased postoperative rescue analgesia requirements [odds ratio (OR), 1.306; 95% confidence interval (CI), 1.031–1.655] [17]. However, very few large scale clinical studies have investigated the relationship between the surgical patient’s preoperative anticipated pain and the actual pain intensity experienced after operations in the general surgical population. Therefore, this clinical observational study aimed to determine the patient characteristics and perioperative factors influencing the subjective anticipated pain intensities in patients scheduled for common elective surgical procedures (specific aim 1); Fig. 1. The anticipated pain scales were also compared with the actual pain intensity experienced and analgesia required by the patients after surgery (specific aim 2); Fig. 1.

## Methods

### Study population and study protocol

**Fig. 2:**
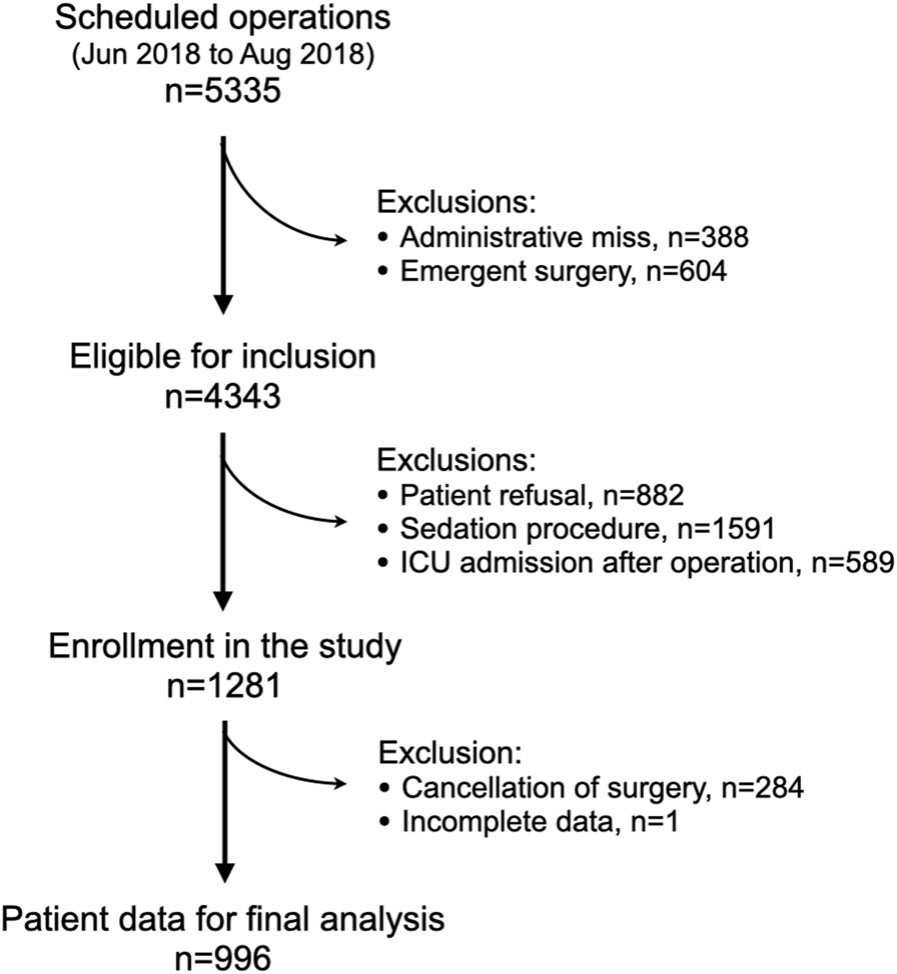
Study flow diagram

This prospective observational study was approved by the ethics committee and the institutional review board of E-Da Hospital, Taiwan (approval number EMRP107018). Consecutive patients who received elective surgery under general or regional anesthesia during June 2018 to August 2018 were included in this study and patients scheduled for emergency operations or those who required postoperative intensive care were excluded (Fig. 2). Patients were invited to voluntarily respond to a quantitative question during their preoperative anesthesia assessment. The patients were asked to rate their highest subjective anticipated postoperative pain intensity (numeric rating scale (NRS) 0–10). After their operations, patients were admitted to the postoperative care unit (PACU). The nurse specialists in the PACU recorded the pain levels by asking the patient’s subjective NRS (1–10) at 15-min intervals. The severity of postoperative pain assessed in the PCAU was defined as low (NRS 1–3) or moderate-to-severe (NRS ≥4). The total analgesic dosages administered in the operating room and in the PACU were also recorded. All anesthetic and surgical interventions administered in this study, including procedures and medications, followed standard clinical practice protocol or physician’s decision. The anesthesia and surgical team members were blinded to the patients’ preoperative anticipated pain scales. The equivalent doses of opioids used during the perioperative period was calculated according to the updated practical opioid rotation and equianalgesic tables [18]. A culturally relevant depression screening questionnaire, the Taiwanese Depression Questionnaire (TDQ), was used to assess for depression in patients admitted to the surgical wards [19]. This 18-item screening tool has a reported sensitivity of 0.89 and a specificity of 0.92 at a cutoff score of 19 for depression screening in the general Taiwanese public [19].

### Statistics

In clinical care, NRS greater than 4 are defined as unacceptable surgical pain levels that required analgesic intervention [20]. Furthermore, patients who reported an expected NRS ≥4 in the preoperative period were associated with a significantly increased risk of postoperative pain up to postoperative day 4 [21]. Therefore, this study categorized the severity of anticipated or the actual postoperative pain surgical pain intensities into low (NRS 1–3) or moderate-to-severe (NRS ≥4), and analyzed the relationships between the moderate-to-severe surgical pain intensities and the clinical variables. Types of surgical procedures that were associated with different levels of expected postoperative pain intensity were graded according to a clinical prediction rule established by Jessen et al. [22]. The risk of developing severe postoperative pain was graded by the invasiveness of the procedure, clinical observation, current practice, and opinions of surgeons and anesthesiologists. A total of 27 groups of surgical procedures were classified into 5 levels, as lowest, low, moderate, high and highest expected pain (supplementary Table 1) [22]. Variance inflation factors (VIF) were computed for the covariates that are potentially affected the preoperative anticipated pain intensities. A VIF of 1.0 indicates that the particular variable of interest is not significantly correlated to the other covariates. The values of NRS for different types of surgery were analyzed using the Kruskal-Wallis test, followed by the Dunn’s post-hoc test. The values of continuous variables were compared using an Wilcoxon rank-sum test or Kruskal-Wallis test, as appropriate. Categorical variables were compared using chi-square or Fisher’s exact test. A stepwise regression model was adopted to evaluate the factors of interest (patient demographic and clinical variables) and the preoperative anticipated pain scales. Statistical significance was accepted at a level of *P* < 0.05. All statistical analyses were performed using the SAS software, version 9.1 (SPSS software, version 24.0 (IBM, Armonk, NY).

## Results

### General outcomes

A total of 996 eligible patients were included in the study, as one patient was excluded due to incomplete data (Fig. 2). Most of the patients underwent a preoperative pain assessment 7 days before surgery (Table 1). Majority of the patients in this study were middle aged and there were no significant gender imbalances (Table 1). More patients received general anesthesia for their procedures and only 1 in 4 patients did not have previous any surgeries (Table 1). Types of operation are listed in Table 1. More than 70% of the patients anticipated moderate-to-severe pain (NRS < 4) after surgery with a mean predicted NRS of 4.9 (Table 1).

**Table 1:** Patient demographical data (*n* = 996)

Characteristics	*n* (%) or mean ± SD
Age (years, mean)	50.9 ± 15.6
Age groups (years)	
0–40	262 (26.3%)
> 40	734 (73.7%)
Gender	
Male	507 (50.9%)
Female	489 (49.1%)
Body mass index (kg/cm^2^, mean)	25.8 ± 5.5
Body mass index (kg/cm^2^)	
< 18.5	41(4.1%)
18.5 ~ 24.9	439 (44.1%)
> 24.9	516 (51.8%)
Educational levels	
Illiteracy	41 (4.1%)
< College or high school	634 (63.7%)
≥ University	321 (32.2%)
Depression (yes)	105 (10.5%)
Surgical history (yes)	736 (73.9%)
Mean anticipated NRS	4.9 ± 2.6
Anticipated moderate-to-severe pain (NRS ≥4)	708 (71.7%)
Types of anesthesia	
General anesthesia	857(86.0%)
Regional anesthesia	139(14.0%)
ASA physical status	
I-II	831(83.4%)
III-V	165(16.6%)
Types of surgery with different expected pain^a^	
Lowest expected pain	108 (10.8%)
Low expected pain	167 (16.8%)
Moderate expected pain	98 (9.8%)
High expected pain	309 (31.0%)
Highest expected pain	314 (31.5%)

*ASA* American Society of Anesthesiologists, *NRS* numeric rating scale; ^a^Types of surgical procedures that were associated with different levels of expected postoperative pain intensity were graded according to a clinical prediction rule established by Jessen and his colleagues [22]

### Patient characteristics and perioperative factors

Table 2 presences the univariate analysis of patient characteristics and perioperative factors that associated with moderate-to-severe anticipated pain before operation. Some of these clinically relevant factors with optimally low values of VIF (supplementary Table 2) were processed for conditional multivariate logistic regression analysis (Table 3). Multivariate analysis showed that female gender was associated with significantly higher anticipated pain intensity with an OR of 1.523 (95% CI) 1.126–2.061; *P* = 0.006) (Table 3). Furthermore, patients who took regular benzodiazepines at bedtime reported significantly higher anticipated pain intensities (AOR 1.670; 95% CI 1.032–2.702, *P* = 0.037) (Table 3). Compared with those who were younger, less patients over 40 years of age anticipated moderate-to-severe surgical-related pain before operation (AOR 0.739; 95% CI 0.518–1.056, *P* = 0.097) (Table 3). Although univariate analysis found that regional anesthesia was associated with significantly higher anticipation for moderate-to-severe surgical pain (Table 2), the effects of different anesthesia techniques (regional vs general) on anticipated moderate-to-severe pain levels were unsignificant in multivariate analysis (Table 3).

**Table 2:** Univariate analysis of the associations between patient’s anticipated pain intensity and the perioperative factors

	Anticipated moderate-to-severe pain^a^		
	AOR	95% CI	*P* value
Gender			
Male	Ref		0.001
Female	1.971	1.488–2.611	
Age (years)			
0–40	Ref		0.003
> 40	0.608	0.439–0.848	
Prior surgical history			
No	Ref		0.266
Yes	0.834	0.607–1.148	
BMI			
< 18.5	Ref		
18.5 ~ 24.9	1.176	0.584–2.382	0.652
> 24.9	0.912	0.454–1.834	0.797
Regular benzodiazepine use at bedtime			
No	Ref		0.035
Yes	1.632	1.035–2.574	
Depression^b^			
No	Ref		0.409
Yes	1.231	0.752–2.017	
Educational levels			
Illiteracy	Ref		
< High school	1.074	0.545–2.119	0.836
≥ University	1.331	0.659–2.691	0.425
ASA physical status			
I-II	Ref		0.114
III-V	0.751	0.526–1.072	
Types of anesthesia			
General anesthesia	Ref		0.046
Regional anesthesia	0.682	0.467–0.994	
Types of surgery with different expected pain^c^			
Lowest	Ref		
Low	2.387	1.452–3.922	0.001
Moderate	3.010	1.687–5.373	< 0.001
High	3.757	2.373–5.948	< 0.001
Highest	4.258	2.678–6.769	< 0.001

*AOR* adjusted odd ratio, *ASA* American Society of Anesthesiologists, *CI* confidence interval, *NRS* numeric rating scale. ^a^Moderate-to-severe anticipated pain was defined as a NRS ≥4. ^b^The presence of depressive symptoms was screened using a culturally relevant depression screening questionnaire, the Taiwanese Depression Questionnaire (TDQ) [19]. ^c^Types of surgical procedures that were associated with different levels of expected postoperative pain intensity were graded according to a clinical prediction rule established by Jessen and his colleagues [22]. A stepwise regression model was used to determine the values of AOR and 95% CI for each associated factor

**Table 3:** Multivariate analysis of the predicting factors for anticipated moderate-to-severe pain

	Anticipated moderate-to-severe pain^*^		
	AOR	95% CI	*P* value
Gender			
Male	Ref		0.006
Female	1.523	1.126–2.061	
Age (years)			
0–40	Ref		0.097
> 40	0.739	0.518–1.056	
Prior surgical history			
No	Ref		0.323
Yes	0.843	0.600–1.184	
Regular benzodiazepine use at bedtime			
No	Ref		0.037
Yes	1.670	1.032–2.702	
ASA physical status			
I-II	Ref		0.201
III-V	0.778	0.530–1.143	
Types of anesthesia			
General anesthesia	Ref		0.870
Regional anesthesia	1.036	0.678–1.583	
Types of surgery with different expected pain^b^			
Lowest	Ref		
Low	2.109	1.268–3.508	0.004
Moderate	2.448	1.349–4.441	0.003
High	3.296	2.046–5.310	< 0.001
Highest	3.411	2.090–5.568	< 0.001

*AOR* adjusted odd ratio, *ASA* American Society of Anesthesiologists, *CI* confidence interval, *NRS* numeric rating scale. ^a^Moderate-to-severe anticipated pain was defined as a NRS ≥4. A stepwise regression model was used to determine the values of AOR and 95% CI for each associated factor. ^b^Types of surgical procedures that were associated with different levels of expected postoperative pain intensity were graded according to a clinical prediction rule established by Jessen and his colleagues [22]

**Fig. 3:**
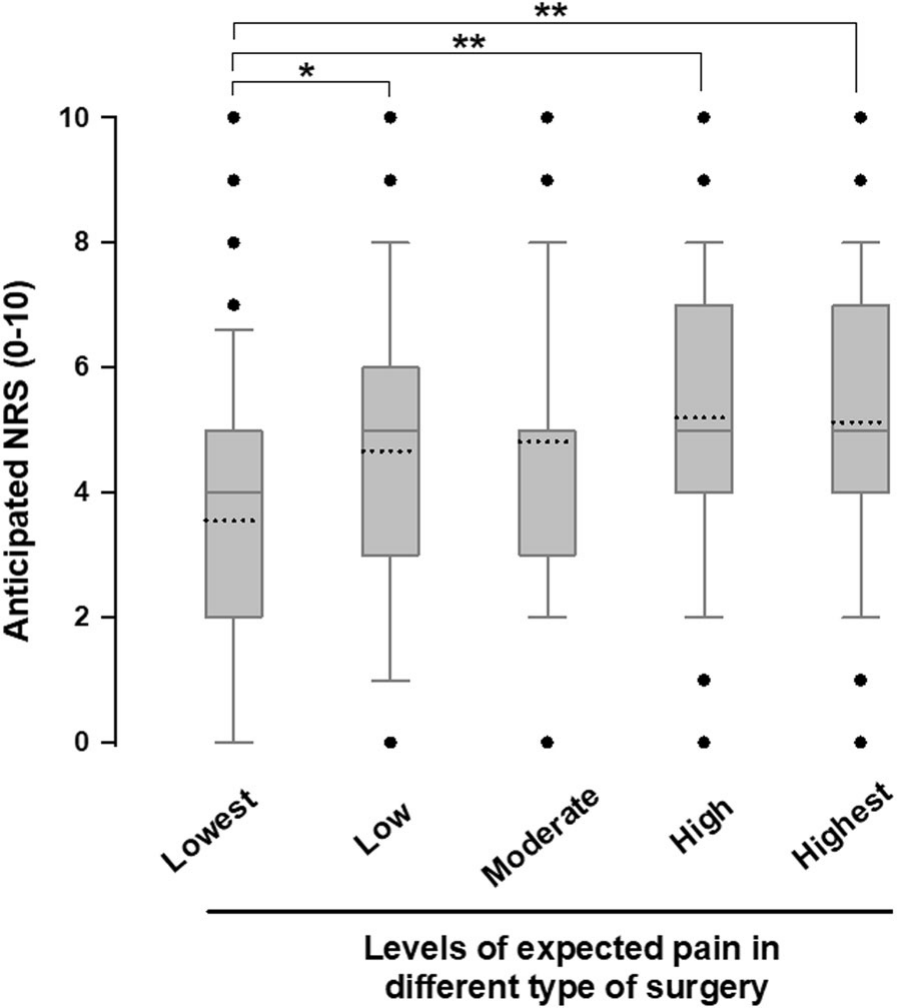
Graphical presentation of relationships between types of the scheduled surgery and patient’s anticipated pain. The invasiveness of surgical procedures graded by a clinical prediction model established by Janssen and his colleageus [22], as types of operation were grouped into the lowest, low, moderate, high and highest expected pain surgery. The median value of anticipated numeric rating scale (NRS) in the lowest expected pain surgery group was significantly increased in comparison to the other groups (*P < 0.05 and **P < 0.001; as analyzed using the Kruskal-Wallis test, followed by the Dunn’s post-hoc test). Results are presented as box-and-whisker plots, in which the horizontal solid lines of boxes indicate the 75th percentile, median and 25th percentile of the distribution, and the upper and lower whiskers indicate the maximal and minimal values. Dotted lines indicate the mean values

There were 27 different surgical procedures, which could be categorized into 5 classes (supplementary Table 1). Patients scheduled for lowest expected pain procedures anticipated of a mean NRS of 3.3 ± 2.5; while the anticipated NRS for those scheduled for highest expected pain procedures were 5.3 ± 2.5 (*P* < 0.001) (Fig. 3). Multivariate analysis suggested that patients scheduled to receive surgical procedures with low to highest expected pain were more likely to anticipate for higher pain intensity at preoperative period than those who scheduled for the lowest expected pain procedures (*P* < 0.05) (Table 3). In addition, there was a linear relationship of increasing intensity of anticipated pain with different classifications of surgical procedures (Fig. 3, Tables 2 and 3).

### Association between preoperative anticipated pain and postoperative pain

Before surgery, 71.2% of patients anticipated moderate-to-severe pain intensity (NRS ≥4) (Table 4), but the actual NRS recorded by PACU nurses showed that 58.2% of patients had adequate pain control (highest NRS < 4) within 1 h after surgery (Table 4). Patients who anticipated high NRS during preoperative period were associated with significantly higher actual NRS in PACU (*P* = 0.007) and also received significantly higher total equivalent opioid doses during the perioperative period (*P* < 0.001) (Table 5).

**Table 4:** Comparison between patient’s preoperative self-anticipated pain and postoperative pain score measured in PACU

Highest pain score at PACU	Preoperative self-anticipated pain score		*P* value =0.005
	NRS < 4	NRS 4–10	
NRS < 4	185 (18.7%)	389 (39.4%)	574 (58.2%)
NRS 4–10	99 (10.0%)	314 (31.8%)	413 (41.8%)
Total patients (*n* = 987)^a^	284 (28.8%)	703 (71.2%)	

*NRS* numeric rating scale, *PACU* post-anesthesia care unit. ^a^A total of 987 datasets were analyzed due to missing of the NRS in the PACU

**Table 5:** Analysis of the associations between patient’s preoperative self-anticipated pain intensity and the actual postoperative pain at PACU or analgesic requirement during perioperative period (*n* = 996)

	Preoperative self-anticipated pain score		*P* value
	NRS < 4	NRS 4–10	
Highest pain score at PACU (NRS)	2.37 ± 1.89	2.77 ± 2.03	0.007
Equivalent dose of opioid (mg) during perioperative period	12.81 ± 8.14	15.04 ± 9.15	< 0.001

*NRS* numeric rating scale, *PACU* post-anesthesia care unit

## Discussion

A major limitation in postoperative pain management has been the fact that a patient’s personal perception of pain may not always be taken into account during preoperative pain counseling. Acute postoperative pain is a subjective and multidimensional experience that is extremely hard to measure and manage optimally. In fact, a previous study found that pre-exposure to a stressed or anxious condition significantly increased the subjective pain perception to a standard noxious stimulation than those who were pre-exposed to a happy condition [23].

Gender is commonly considered as a strong predictor for pain perception and analgesic requirements after surgery [24, 25]. However, some systematic reviews have not found gender to be an independent predictor for postoperative pain levels or analgesic requirements [14]. The results of our survey suggest that female patients anticipated significantly higher pain levels preoperatively than male patients, the difference remained statistically significant following a multivariate regression analysis with an odds ratio of 1.523 (95% CI 1.126–2.061). These results support the findings of numerous previous studies [26–28]. The univariate analysis also found that patients over 40 years of age anticipated a lesser degree of surgery-related pain during their preoperative assessments as compared to those who were younger. This observation is consistent with previous prospective observational studies in patients receiving breast surgery, indicating that age had a negative impact on the prediction of acute postoperative pain [16, 29], and previous studies also found that the elderly are usually associated with less preoperative anxiety [30, 31]. However, difference in age groups became an insignificant factor for increased anticipation of moderate-to-severe pain during preoperative period in multivariate analysis, probably due to the neutralization effect of surgical types with different expected pain levels in these two age groups.

Previous studies have suggested that patients with psychosomatic and behavioral disorders (e.g. *major* depression, insomnia, and catastrophizing pain) can have a decreased tolerances for postoperative pain [32–35]. Our study has found that regular benzodiazepine use at bedtime is an independent risk factor for high anticipated postoperative pain intensity during preoperative assessments. In addition to hypnosis, benzodiazepines are also commonly used to manage anxiety and other anxiety-related disorders. However, we did not specify the clinical indications for the regular use of benzodiazepines for individual patients. According to our questionnaire design, the use of benzodiazepines at bedtime was more likely considered as hypnotic agents to improve sleeping quality at night, rather than surrogate indicators for anxiety or other psychosomatic disorders. Furthermore, no differences were found in preoperative pain anticipation between surgical patients with and without depression, which was screened by the Taiwanese Depression Questionnaire during preoperative assessment. This study also did not find significant effects of other patient characteristic variables, such as educational levels, marital and socioeconomic status on the anticipation of surgical pain intensity.

Classification of type of surgery has been shown as a clinical meaningful predictor for prediction of acute postoperative pain, as the invasiveness and incision size of surgical procedure correlate with the anticipated pain intensity [22]. We used the clinical prediction rule established by Janssen et al., in which types of surgery were graded from the lowest to the highest expected pain procedures [22]. Our analysis showed a clear positive relationship between type of operation and patient’s anticipated pain intensity, suggesting that the invasiveness and complexity of procedure affects patients’ anticipated perception of surgical-related pain in the preoperative period [14, 22]. Previous studies also indicate that anesthetic techniques play a major role in the risk of developing severe acute postoperative pain, as the odds ratio of NRS > 4 was higher in patients receiving only general anesthesia without regional block techniques immediately after operation and on postoperative day 2 [21]. Consistently, our univariate analysis suggested that the proposed administration of regional blocks significantly reduced patient concerns regarding postoperative pain. However, techniques of anesthesia (regional or general anesthesia) were not significantly associated with the anticipation of moderate-to-severe pain in multivariate analysis, which was also most likely affected by differences in types of surgery.

Preoperative anticipated pain intensity was compared with the highest postoperative pain intensity recorded in PACU and the total equivalent dose of opioids prescribed perioperatively. Our analysis found that patients who anticipated moderate-to-severe pain intensity before operations were associated with significantly higher actual pain scores in the PACU and also required significantly higher doses of analgesics during the perioperative period compared to those who reported a lower preoperative pain anticipation. In current practice, anesthesiologists are more likely to prescribe postoperative analgesics based on the type and duration of the operation rather than the patient’s subjective perception of pain [36, 37]. Our results suggest that patient’s self-anticipated pain intensity may provide complementary clinical considerations for adequate management of acute pain after surgery.

After extensively reviewing 48 studies, Ip et al. identified several independent perioperative factors for predicting actual levels of postoperative pain and analgesic usage [14]. These predictive factors include the presence of preoperative pain, anxiety, age, and type of surgery (i.e. major joint, thoracic, and open abdominal surgery) and are associated with higher postoperative pain scores. Surgery type, age, and psychological distress were found to be significant predictors of analgesic usage. Ip and colleagues’ systematic review found that gender had a neutral effect on postoperative pain levels and analgesic requirements, but the results of our study indicated that females anticipated more postoperative pain preoperatively. This major discrepancy could be due to the general understanding that female patients can react more emotionally to physical distress, but the distress is no less authentic and they are not less ill than the male patients [38–40].

The results of this study must be interpreted in light of several limitations. Firstly, patients were invited to voluntarily rate the anticipated pain intensity during their preoperative anesthesia assessment. Therefore, the knowledge, educational levels and motives of the individual patient might impact the response to the quantitative question. Secondly, patients’ preoperative psychological conditions are routinely assessed using a culturally relevant depression screening questionnaire, the Taiwanese Depression Questionnaire (TDQ) in our hospital. This short questionnaire were designed to be simple and practical so that it could be applied to the general population in a time-efficient manner. The comprehensive versions for diagnosing depression and chronic insomnia were not used in this study. Furthermore, the use of a structured self-rating Pain Sensitivity Questionnaire may also provide higher sensitivity to predict the development of acute postoperative pain [16, 41]. Thirdly, several potential predicting factors, such as patient’s pain catastrophism, pain sensitivity, preoperative opioid intake, full history of past surgeries and traumas, and ethnicity were not determined in this study. Although total equianalgesic doses of opioid administered during perioperative period were calculated, the use of non-opioid analgesics were not taken into account for the overall surrogate indicator for postoperative pain. Lastly, our results were not generalized to critically ill patients who were scheduled for postoperative intensive care or emergent surgery.

## Conclusion

Our study demonstrated that female gender, regular benzodiazepine use at bedtime and who scheduled to receive more invasive surgical procedures anticipate significantly higher pain intensity before surgery, and they are associated with higher actual pain scores and increased analgesic requirements during the perioperative period. Therefore, these patients may require additional assessments and pain management counseling during their pre-anesthesia consultation. Appropriate preoperative counseling for analgesic control (especially the introduction of multimodal analgesia) and the management of unnecessary anticipated pain levels could improve the quality of anesthesia delivery and patient perioperative satisfaction.

## Supplementary Information

**Additional file 1: Supplementary table 1.** Surgical procedures with different levels of expected pain [19]

**Additional file 2: Supplementary table 2.** Variance inflation factors (VIF) for the degree of multicollinearity among patient’s characteristic and surgical-related variables that associated with preoperative anticipated pain. ASA: American Society of Anesthesiologists. *A total of 27 groups of surgical procedures were classified into 5 levels, as lowest, low, moderate, high and highest expected pain [22]

## Data Availability

The datasets used and analyzed during the current study are available from the corresponding author upon reasonable request.

## Abbreviations

AOR: Adjusted odds ratio
ASA PS: American Society for Anesthesiologist physical statuses
BMI: Body mass index
CI: Confidence interval
ERAS: Enhanced recovery after surgery
MMA: Multimodal analgesia
NRS: Numeric rating scale
PACU: Postanesthesia care unit
TDQ: The Taiwanese Depression Questionnaire
VAS: Visual analogue scale

## References

[1] Kehlet H, Jensen TS, Woolf CJ (2006). Persistent postsurgical pain: risk factors and prevention. Lancet..

[2] Macrae WA (2001). Chronic pain after surgery. Br J Anaesth.

[3] Macrae WA (2008). Chronic post-surgical pain: 10 years on. Br J Anaesth.

[4] Bechert K, Abraham SE (2009). Pain management and wound care. J Am Col Certif Wound Spec.

[5] Woo KY (2012). Exploring the effects of pain and stress on wound healing. Adv Skin Wound Care.

[6] Lynch EP, Lazor MA, Gellis JE, Orav J, Goldman L, Marcantonio ER (1998). The impact of postoperative pain on the development of postoperative delirium. Anesth Analg.

[7] Breivik H, Borchgrevink PC, Allen SM, Rosseland LA, Romundstad L, Hals EK, Kvarstein G, Stubhaug A (2008). Assessment of pain. Br J Anaesth.

[8] Apfelbaum JL, Chen C, Mehta SS, Gan TJ (2003). Postoperative pain experience: results from a national survey suggest postoperative pain continues to be undermanaged. Anesth Analg.

[9] El-Aqoul A, Obaid A, Yacoub E, Al-Najar M, Ramadan M, Darawad M (2018). Factors associated with inadequate pain control among postoperative patients with Cancer. Pain Manag Nurs.

[10] Meyer LA, Lasala J, Iniesta MD, Nick AM, Munsell MF, Shi Q, Wang XS, Cain KE, Lu KH, Ramirez PT (2018). Effect of an enhanced recovery after surgery program on opioid use and patient-reported outcomes. Obstet Gynecol.

[11] Gan TJ (2017). Poorly controlled postoperative pain: prevalence, consequences, and prevention. J Pain Res.

[12] Pavlin DJ, Sullivan MJ, Freund PR, Roesen K (2005). Catastrophizing: a risk factor for postsurgical pain. Clin J Pain.

[13] Banka TR, Ruel A, Fields K, YaDeau J, Westrich G (2015). Preoperative predictors of postoperative opioid usage, pain scores, and referral to a pain management service in total knee arthroplasty. HSS J.

[14] Ip HY, Abrishami A, Peng PW, Wong J, Chung F (2009). Predictors of postoperative pain and analgesic consumption: a qualitative systematic review. Anesthesiology..

[15] Sipila RM, Haasio L, Meretoja TJ, Ripatti S, Estlander AM, Kalso EA (2017). Does expecting more pain make it more intense? Factors associated with the first week pain trajectories after breast cancer surgery. Pain..

[16] Rehberg B, Mathivon S, Combescure C, Mercier Y, Savoldelli GL (2017). Prediction of acute postoperative pain following breast Cancer surgery using the pain sensitivity questionnaire: a cohort study. Clin J Pain.

[17] Pinto PR, McIntyre T, Fonseca C, Almeida A, Araújo-Soares V (2013). Pre- and post-surgical factors that predict the provision of rescue analgesia following hysterectomy. Eur J Pain.

[18] Treillet E, Laurent S, Hadjiat Y (2018). Practical management of opioid rotation and equianalgesia. J Pain Res.

[19] Lee Y, Yang MJ, Lai TJ, Chiu NM, Chau TT (2000). Development of the Taiwanese depression questionnaire. Chang Gung Med J.

[20] Gerbershagen HJ, Rothaug J, Kalkman CJ, Meissner W (2011). Determination of moderate-to-severe postoperative pain on the numeric rating scale: a cut-off point analysis applying four different methods. Br J Anaesth.

[21] Sommer M, de Rijke JM, van Kleef M, Kessels AG, Peters ML, Geurts JW, Patijn J, Gramke HF, Marcus MA (2010). Predictors of acute postoperative pain after elective surgery. Clin J Pain.

[22] Janssen KJ, Kalkman CJ, Grobbee DE, Bonsel GJ, Moons KG, Vergouwe Y (2008). The risk of severe postoperative pain: modification and validation of a clinical prediction rule. Anesth Analg.

[23] Lefebvre JC, Jensen MP (2019). The relationships between worry, happiness and pain catastrophizing in the experience of acute pain. Eur J Pain.

[24] Cepeda MS, Carr DB (2003). Women experience more pain and require more morphine than men to achieve a similar degree of analgesia. Anesth Analg.

[25] Chia YY, Chow LH, Hung CC, Liu K, Ger LP, Wang PN (2002). Gender and pain upon movement are associated with the requirements for postoperative patient-controlled iv analgesia: a prospective survey of 2,298 Chinese patients. Can J Anaesth.

[26] Palmeira CC, Ashmawi HA, Posso IP (2011). Sex and pain perception and analgesia. Rev Bras Anestesiol.

[27] Unruh AM (1996). Gender variations in clinical pain experience. Pain..

[28] Samulowitz A, Gremyr I, Eriksson E, Hensing G. "Brave men" and "emotional women": a theory-guided literature review on gender Bias in health care and gendered norms towards patients with chronic pain. Pain Res Manag. 2018:6358624. 10.1155/2018/6358624 PMC5845507 29682130

[29] Schreiber KL, Zinboonyahgoon N, Xu X, Spivey T, King T, Dominici L, Partridge A, Golshan M, Strichartz G, Edwards RR (2019). Preoperative psychosocial and psychophysical phenotypes as predictors of acute pain outcomes after breast surgery. J Pain.

[30] Celik F, Edipoglu IS (2018). Evaluation of preoperative anxiety and fear of anesthesia using APAIS score. Eur J Med Res.

[31] Erkilic E, Kesimci E, Soykut C, Doger C, Gumus T, Kanbak O (2017). Factors associated with preoperative anxiety levels of Turkish surgical patients: from a single center in Ankara. Patient Prefer Adherence.

[32] Taenzer P, Melzack R, Jeans ME (1986). Influence of psychological factors on postoperative pain, mood and analgesic requirements. Pain..

[33] De Cosmo G, Congedo E, Lai C, Primieri P, Dottarelli A, Aceto P (2008). Preoperative psychologic and demographic predictors of pain perception and tramadol consumption using intravenous patient-controlled analgesia. Clin J Pain.

[34] Papaioannou M, Skapinakis P, Damigos D, Mavreas V, Broumas G, Palgimesi A (2009). The role of catastrophizing in the prediction of postoperative pain. Pain Med.

[35] Khan RS, Skapinakis P, Ahmed K, Stefanou DC, Ashrafian H, Darzi A, Athanasiou T (2012). The association between preoperative pain catastrophizing and postoperative pain intensity in cardiac surgery patients. Pain Med.

[36] Gerbershagen HJ, Aduckathil S, van Wijck AJ, Peelen LM, Kalkman CJ, Meissner W (2013). Pain intensity on the first day after surgery: a prospective cohort study comparing 179 surgical procedures. Anesthesiology..

[37] Shoar S, Esmaeili S, Safari S (2012). Pain management after surgery: a brief review. Anesth Pain Med.

[38] Colameco S, Becker LA, Simpson M (1983). Sex bias in the assessment of patient complaints. J Fam Pract.

[39] Etherton J, Lawson M, Graham R (2014). Individual and gender differences in subjective and objective indices of pain: gender, fear of pain, pain catastrophizing and cardiovascular reactivity. Appl Psychophysiol Biofeedback.

[40] Vambheim SM, Øien RA (2017). Sex differences in fear of pain: item-level analysis of the fear of pain questionnaire III. J Pain Res.

[41] Ruscheweyh R, Marziniak M, Stumpenhorst F, Reinholz J, Knecht S (2009). Pain sensitivity can be assessed by self-rating: development and validation of the pain sensitivity questionnaire. Pain..

