# Supplementary material for: Characterization of self-anticipated pain score prior to elective surgery - a prospective observational study
Source: BMC Anesthesiol. 2021 Mar 19;21:85. doi: 10.1186/s12871-021-01303-y (PMC7977573; doi:10.1186/s12871-021-01303-y)
Supplement: Supplementary file 1 — Additional file 1: Supplementary table 1. Surgical procedures with different levels of expected pain [19] [file 12871_2021_1303_MOESM1_ESM.docx]

**Supplementary table 1.** **Surgical procedures with different levels of expected pain [19]**

| Levels of expected pain | Type of surgery |
| --- | --- |
| Lowest expected pain | Endoscopic urology, testical surgery (orchidopexy, biopsy, prosthesis implantation, vasoepididymostomy, testis-scrotum exploration), eye surgery (including strabismus) |
| Low expected pain | Pharyngo- and laryngoscopy plus biopsy, ear nose throat surgery, diagnostic laparoscopy, gynecologic surgery (non-abdominal non-laparoscopic), minor rectal surgery, oral soft tissue surgery, carotid endarterectomy |
| Moderate expected pain | Skin surgery or lymph node biopsy, peripheral vascular procedure (including varicose veins), minor breast surgery, procedures on muscle and/or ligaments of extremities, upper abdominal surgery with epidural (including hepato-biliary, esophageal, pancreatic and intestinal surgery) |
| High expected pain | Major breast surgery, bone procedure (including cranial/facial, oral, spine, orthopedic/traumatology procedure on clavicle extremities, hip and pelvis), instrumentation or remove of instrumentation (spine, hip, jaw/denture, hand/wrist, clavicle, elbow, ankle/foot or knee), arthroscopy of shoulder, hip/pelvis and extremities, procedure for abdominal wall herniation, nephrectomy |
| Highest expected pain | Therapeutic laparoscopic procedure (cholecystectomy, gynecologic laparoscopy and other therapeutically laparoscopy), intrabdominal surgery without epidural (colon, bladder, prostate, vascular and gynecological surgery), tonsillectomy (>16 years), herniated disc surgery, bone procedure including shoulder, thoracotomies, elbow, ankle/foot (excluding instrumentation or remove of instrumentation), thyroid procedures, peripheral nerve reconstruction, vaginal hysterectomy. |
