# Supplementary material for: Characterization of self-anticipated pain score prior to elective surgery - a prospective observational study
Source: BMC Anesthesiol. 2021 Mar 19;21:85. doi: 10.1186/s12871-021-01303-y (PMC7977573; doi:10.1186/s12871-021-01303-y)
Supplement: Supplementary file 2 — Additional file 2: Supplementary table 2. Variance inflation factors (VIF) for the degree of multicollinearity among patient’s characteristic and surgical-related variables that associated with preoperative anticipated pain. ASA: American Society of Anesthesiologists. *A total of 27 groups of surgical procedures were classified into 5 levels, as lowest, low, moderate, high and highest expected pain [22] [file 12871_2021_1303_MOESM2_ESM.docx]

**Supplementary table 2.** Variance inflation factors (VIF) for the degree of multicollinearity among patient’s characteristic and surgical-related variables that associated with preoperative anticipated pain

| Variables associated with anticipated pain | VIF | |
| --- | --- | --- |
| Gender | | 1.134 |
| Age (years) | | 1.228 |
| Prior surgical history | | 1.054 |
| BMI | | 1.019 |
| Regular benzodiazepine use at bedtime | | 1.053 |
| Depression | | 1.019 |
| Educational levels | | 1.219 |
| ASA physical status | | 1.075 |
| Types of anesthesia | | 1.073 |
| Types of surgery defined by expected pain levels* | | 1.158 |

ASA: American Society of Anesthesiologists. *A total of 27 groups of surgical procedures were classified into 5 levels, as lowest, low, moderate, high and highest expected pain [22].
